# Supplementary material for: Identification of photoperiod-induced specific miRNAs in the adrenal glands of Sunite sheep (Ovis aries)
Source: Front Vet Sci. 2022 Jul 22;9:888207. doi: 10.3389/fvets.2022.888207 (PMC9354845; doi:10.3389/fvets.2022.888207)
Supplement: Supplementary file 1 [file Data_Sheet_1.ZIP › Supplementary file/miRDeep2 file.zip!/miRDeep2 file/Novel_Structure/LP42A1/Novel_644.pdf]

Provisional ID	:	NC_019473.2_25517
Score total	:	2.8
Score for star read(s)	:	-1.3
Score for read counts	:	0
Score for mfe	:	2.5
Score for randfold	:	1.6
Score for cons. seed	:	
Total read count	:	38
Mature read count	:	36
Loop read count	:	0
Star read count	:	2

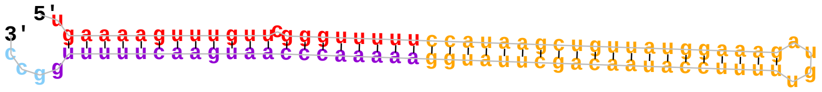

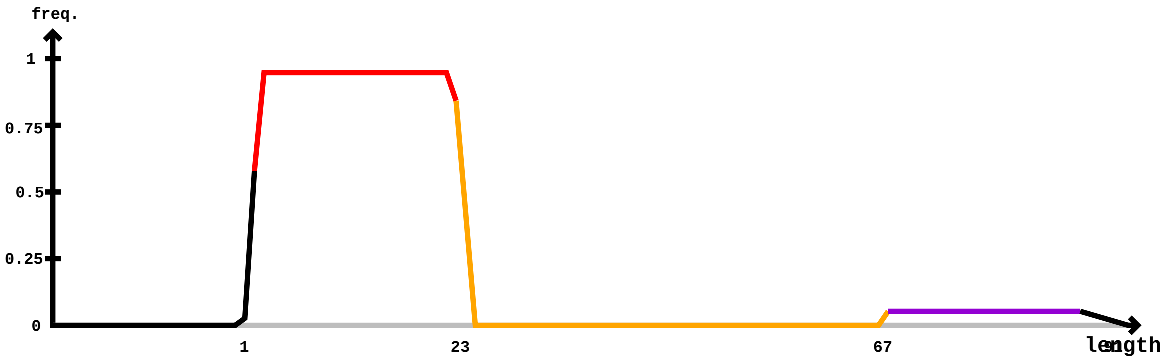


```
                    Mature                                                      Star
5'- cccacagacacugggguuggugaaaaguuuguucggguuuuuccauaagcuguuauggaaagauguuuuuccauaacagcuuauggaaaaacccaaugaacuuuuuggccaa   -3'    obs
    cccacagacacugggguuggugaaaaguuuguucggguuuuuccauaagcuguuauggaaagauguuuuuccauaacagcuuauggaaaaacccaaugaacuuuuuggccaa          exp
    ((((.......))))(((((.((((((((((.(((((((((((((((((((((((((((((....)))))))))))))))))))))))))))))))))))))))..)))))    reads     mm        sample
    ..................gugaaaaguuuguucggguuuuu......................................................................    1         0         seq
    ...................ugaaaaguuuguucggguuuu.......................................................................    3         0         seq
    ...................ugaaaaguuuguucggguuuuu......................................................................    18        0         seq
    ....................Aaaaaguuuguucggguuuu.......................................................................    1         1         seq
    ....................gaaaaguuuguucggguuuuu......................................................................    11        0         seq
    ....................Aaaaaguuuguucggguuuuu......................................................................    2         1         seq
    .....................................................................................aaaaacccaaugaacuuuuug.....    2         0         seq
```
